# Automatic classification of ligneous leaf diseases via hierarchical vision transformer and transfer learning

**DOI:** 10.3389/fpls.2023.1328952

**Published:** 2024-01-12

**Authors:** Dianyuan Han, Chunhua Guo

**Affiliations:** Media and Communications College of Weifang University, Weifang, Shandong, China

**Keywords:** precision agriculture, transformer, neural networks, machine vision, transfer learning

## Abstract

**Background:**

Identification of leaf diseases plays an important role in the growing process of different types of plants. Current studies focusing on the detection and categorization of leaf diseases have achieved promising outcomes. However, there is still a need to enhance the performance of leaf disease categorization for practical applications within the field of Precision Agriculture.

**Methods:**

To bridge this gap, this study presents a novel approach to classifying leaf diseases in ligneous plants by offering an improved vision transformer model. The proposed approach involves utilizing a multi-head attention module to effectively capture contextual information about the images and their classes. In addition, the multi-layer perceptron module has also been employed. To train the proposed deep model, a public dataset of leaf disease is exploited, which consists of 22 distinct kinds of images depicting ligneous leaf diseases. Furthermore, the strategy of transfer learning is employed to decrease the training duration of the proposed model.

**Results:**

The experimental findings indicate that the presented approach for classifying ligneous leaf diseases can achieve an accuracy of 85.0% above.

**Discussion:**

In summary, the proposed methodology has the potential to serve as a beneficial algorithm for automated detection of leaf diseases in ligneous plants.

## 1 Introduction

The occurrence of leaf diseases in plants holds significant relevance in the field of plant pathology. Severe leaf disease can have detrimental effects on plants, including leaf drying and hindered bud formation. It can weaken the health of the plant and worsen the susceptibility to other diseases Kai et al. (2011) *;* Bo et al. (2019) *;* Xu et al. (2020) *;* Wang et al. (2021; 2022). In addition, the occurrence of fruit leaf disease can lead to a decline in both the quantity and quality of fruits, as well as increase the vulnerability of nearby plants to infection. Given the strong reliance of the economy on agricultural productivity, the impact of the leaf disease on the environment becomes particularly significant if preventive measures are not implemented in a timely manner. Therefore, the prompt identification of diseases affecting fruit leaves is crucial for human well-being Patil et al. (2017); Afzaal et al. (2021); Mahum et al. (2022). In general, the identification and categorization of leaf diseases have predominantly depended on the human visual system, which is error prone, is time-consuming and labor-intensive. Hence, the implementation of automated leaf disease classification is imperative in the context of fruit production for mitigating both the production and economic losses Sneha and Bagal (2019); JencyRubia and BabithaLincy (2021); Y et al. (2022).

In recent decades, there has been a significant surge in the utilization of machine learning-based algorithms for addressing leaf disease categorization problems. Numerous machine vision algorithms have been proposed to classify illnesses affecting plant leaves. In the study conducted by Singh and Misra (2017), the authors proposed an image segmentation method for the automatic identification and classification of plant leaf diseases, specifically focusing on the minor leaf disease common in pine trees within the United States. The researchers investigated the utilization of several classifier algorithms for the purpose of identifying plant leaf disease. A system for automatic detection of plant disease using image processing techniques was proposed by the authors Mounika and Bharathi (2020). The approach was used for calculation of textural data pertaining to illnesses affecting plant leaves. In their work, Kulkarni and Sapariya (2021) proposed a method to automatically detect and classify leaf illnesses, which encompasses many stages, including image gathering, image pre-processing, segmentation, and classification. In their study, Reddy et al. (2021) employed Support Vector Machine (SVM) and Random Forest algorithms for the purpose of detecting illnesses in leaves. This study compared assessment measures, such as Root Mean Square Error (RMSE), Peak Signal Noise Ratio (PSNR), for the diseaseaffected regions of the leaves to assess their potential impact on agricultural output.

In recent years, deep learning has gained significant interest due to its remarkable achievements in many domains, such as natural language processing (NLP) and machine vision. Consequently, there have been additional advancements in the field of plant leaf disease categorization by the utilization of deep learning models. Liu et al. (2017) introduced a methodology for detecting apple leaf diseases utilizing deep convolutional neural networks (CNNs). The model reported in this study is capable of generating an ample number of diseased images with a deep learning model, AlexNet. The study utilized a dataset including 13,689 images depicting various apple leaf illnesses. The CNN model developed in this research was trained to accurately classify four types of apple leaf diseases. In the study conducted by Anagnostis et al. (2020), a resilient CNN model was developed to address the timely identification of anthracnose, a prevalent fungal disease that affects numerous tree species globally. This model was to use to classify images of plant leaves as either infected or uninfected by anthracnose. The researchers acquired a dataset consisting of grayscale and RGB images. Then, they utilized a rapid Fourier transform to extract characteristics from the images. Finally, to implement the classification task, they employed a CNN model. To effectively identify olive leaf disease, Ksibi et al. (2022) proposed the utilization of ResNet50 and MobileNet models for image feature extraction, employing the technique of feature concatenation. To train the deep learning models employed in this investigation, a dataset including 5,400 images of olive leaves was utilized. These images were acquired from an olive grove using an unmanned aerial vehicle (UAV) equipped with a camera. In their study, Devi et al. (2022) proposed a methodology for the prediction and classification of corn leaf disease. The authors employed transfer learning and the Alexnet model, leveraging the Adaptive Moment Estimation (ADAM) optimizer and the Stochastic Gradient Descent with momentum (SGDM) mechanism. The model was trained and evaluated using a dataset consisting of 5,300 images, which were categorized into four different types: healthy, blight, common rust, and gray leaf spot. Yao et al. (2022) conducted a study focusing on the identification of kiwifruit leaf disease. They developed a publicly available dataset while using the YOLOX target detection algorithm to mitigate the influence of environmental elements. The study of Yu et al. (2022) introduced a method for efficiently detecting soybean illnesses. It leverages a residual attention network (RANet) model. This study included the incorporation of three types of soybean leaf spot diseases, namely soybean brown leaf spot, soybean frog eye leaf spot, and soybean phyllosticta leaf spot, into the dataset. The OTSU algorithm was utilized to pre-process the initial images for eliminating the surrounding features. Additionally, the image dataset was augmented by the application of image enhancement algorithms. Additionally, the residual attention layer was constructed by integrating attention processes into a ResNet18 model.

The majority of the preceding approaches in the field of leaf disease classification have predominantly employed CNN architectures. Regrettably, the CNN-based models have limitations due to the local receptive field inside the convolutional modules. This characteristic directs attention towards the surrounding region in an image, perhaps overlooking the connections between distant pixels. In contrast, the transformer is renowned for its utilization of an attention mechanism to effectively capture and represent the extensive inter-dependencies within the data samples. The successful performance of transformer in NLP tasks has resulted in its integration and use in the field of computer vision Liu et al. (2021). For instance, the work conducted by Qian et al. (2022) introduced a novel strategy for classifying maize leaf diseases using a vision transformer-based method. The authors of the study also gathered RGB images from publicly available databases and experimental fields, classifying them into four distinct categories: southern corn leaf blight, gray leaf spot, southern corn rust, and healthy specimens. Nevertheless, the vision transformer model proposed in this study might provide challenges when used to high-resolution images due to the quadratic computational complexity of the self-attention mechanism in relation to image resolution. Furthermore, the original vision transformer necessitates a substantial allocation of memory capacity and processing resources.

Taking the aforementioned research into consideration, we propose a hierarchical vision transformerbased approach by employing transfer learning strategy, for classifying leaf diseases of ligneous plants. The hierarchical design in the proposed vision transformer yields notable reductions in computational resource requirements and the number of weighting parameters for the vision transformer. Furthermore, this work utilizes the weighting factors that were pre-trained on the dataset ImageNet Russakovsky et al. (2014). To assess the effectiveness of the suggested methodology, a subset of a publicly available dataset was utilized. This subset comprises a total of 22 types of ligneous leaf images. Furthermore, a series of comparative tests were carried out to evaluate the performance of the suggested methodology as well as the state-of-the-art methods. The experimental findings provide evidence that the suggested methodology outperforms the state-of-the-art techniques in terms of accuracy, precision, recall, and, F1 score.

In general, the contributions of this study include:

- A leaf disease classification pipeline is proposed. The proposed model primarily consists of a hierarchical vision transformer.
- The presented vision transformer model comprises of two channels, which are used to extract the features from the original leaf images and the edges in the corresponding images, respectively.
- The experimental findings prove the superiority of the proposed methodology over the state-of-the-art algorithms.

The subsequent sections of this article are structured in the following manner. Section 2 presents an elaborate exposition of the suggested transformer concept. Section 3 provides a detailed account of the experimental methodology employed in this study, as well as the subsequent findings and their analysis. Finally, The study concludes at Section 4.

## 2 Methodology

### 2.1 Dataset collection and pre-processing

The dataset utilized in this research is sourced from the publicly accessible plant dataset of AI Challenger 2018 Wu et al. (2017), which has a total of 10 plant specimens, each classified into one of 27 categories representing either leaf diseases or healthy conditions. In a systematic manner, a total of 61 image classes have been categorized into distinct groups based on species, pest species, and severity levels. The objective of this work is to categorize diseases affecting ligneous fruit leaves. Therefore, only the leaves that were affected by diseases were selected from the dataset for the purposes of training and validation. In this study, a total of 22 categories of images depicting leaf diseases were included in the dataset. These categories encompassed both sick leaves and healthy leaves.

**Figure 1:**
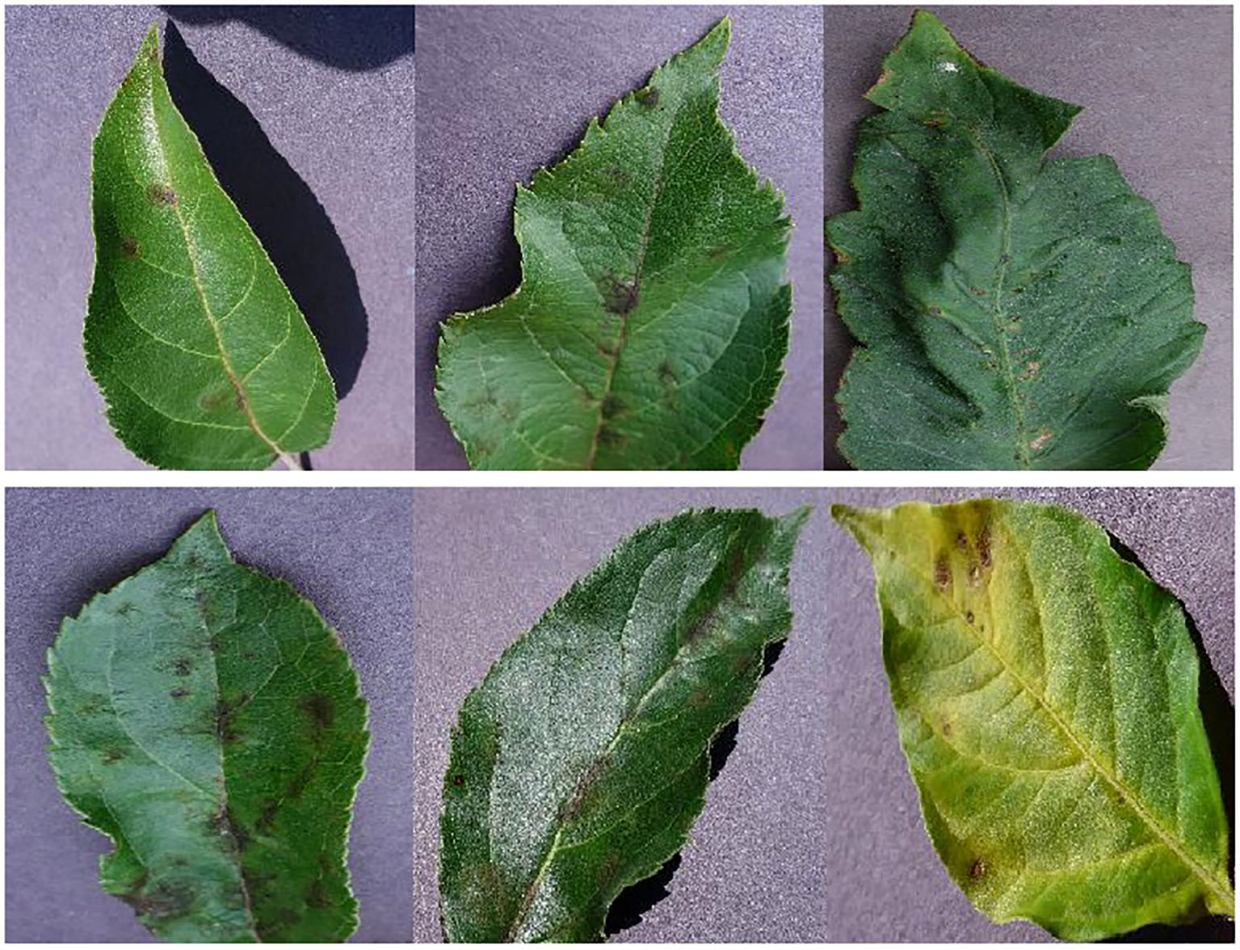
A collection of sample images depicting various types of leaf diseases. The leaves in the top row exhibit signs of good health. The leaves exhibiting signs of illness are seen in the bottom row.

As seen in **Figure 1**, the training set comprises 11,603 images, whereas the testing set consists of 1,668 images. These images are categorized into 22 distinct classes. Furthermore, the dataset includes a collection of example images, as seen in **Figure 1**. These images encompass both healthy and sick leaves.

In this study, the utilization of transfer learning is employed to improve the performance of the proposed approach, taking inspiration from the work of Chen et al. (2020). To achieve this, the proposed model is initially trained on the ImageNet dataset Russakovsky et al. (2014), considering the relatively small size of the presented image dataset. In addition, the images are resized into a uniform dimension of 224×224 to minimize the computing resources needed during the training phase. Moreover, the present study employs a set of data augmentation techniques to increase the number of image samples, which can further enhance the generalization of the proposed model and mitigate the risk of over-fitting during the training process. These techniques include RandomFlip, Color Jitter, Cutmix Yun et al. (2019), and Mixup Zhang et al. (2017).

### 2.2 Overall framework

**Figure 2:**
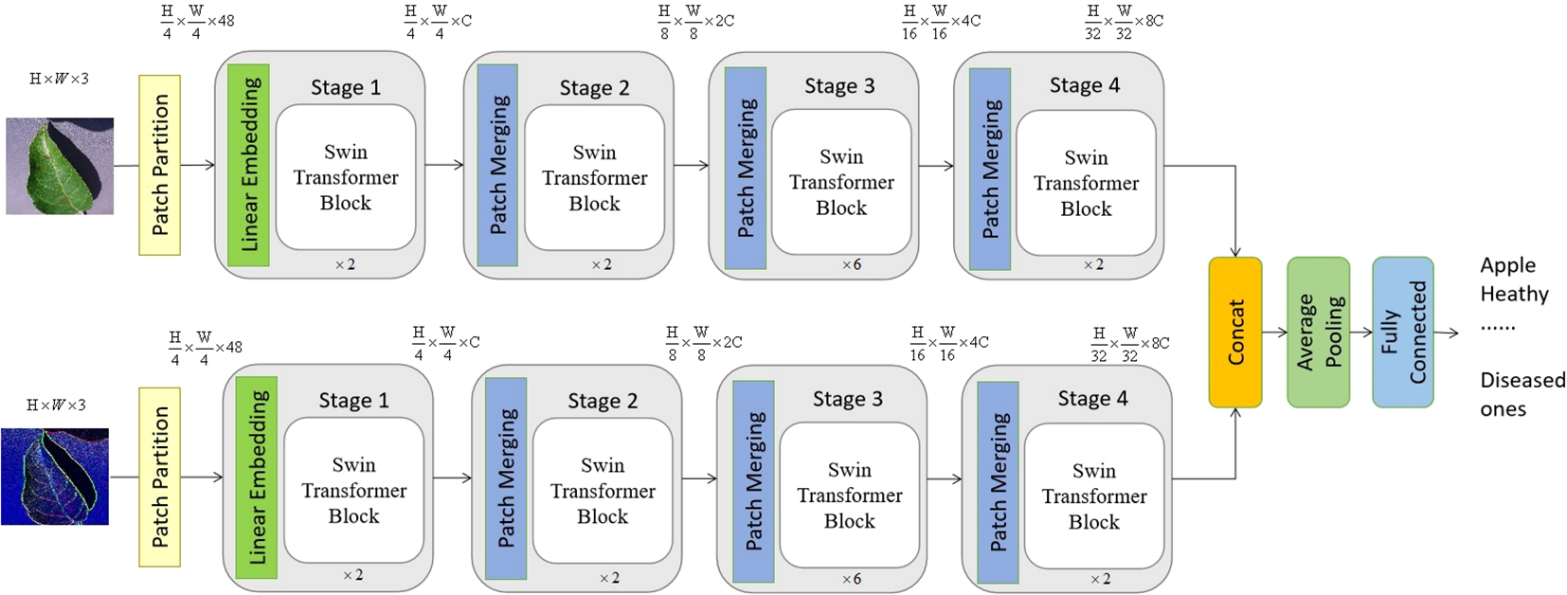
The suggested model consists of a two-channel swin vision transformer, which exhibits a certain overall structure. The top channel of the proposed model receives an initial image as its input, while the lower channel gets the edge information of the original image as its input. It is worth noting that the value of C, which is equal to 96, might vary depending on the architecture of the model.

The proposed vision transformer model is provided in **Figure 2**, which is a typical two-channel swin vision transformer Liu et al. (2021) model, and there is no weighting parameter sharing between these two channels.

**Figure 3:**
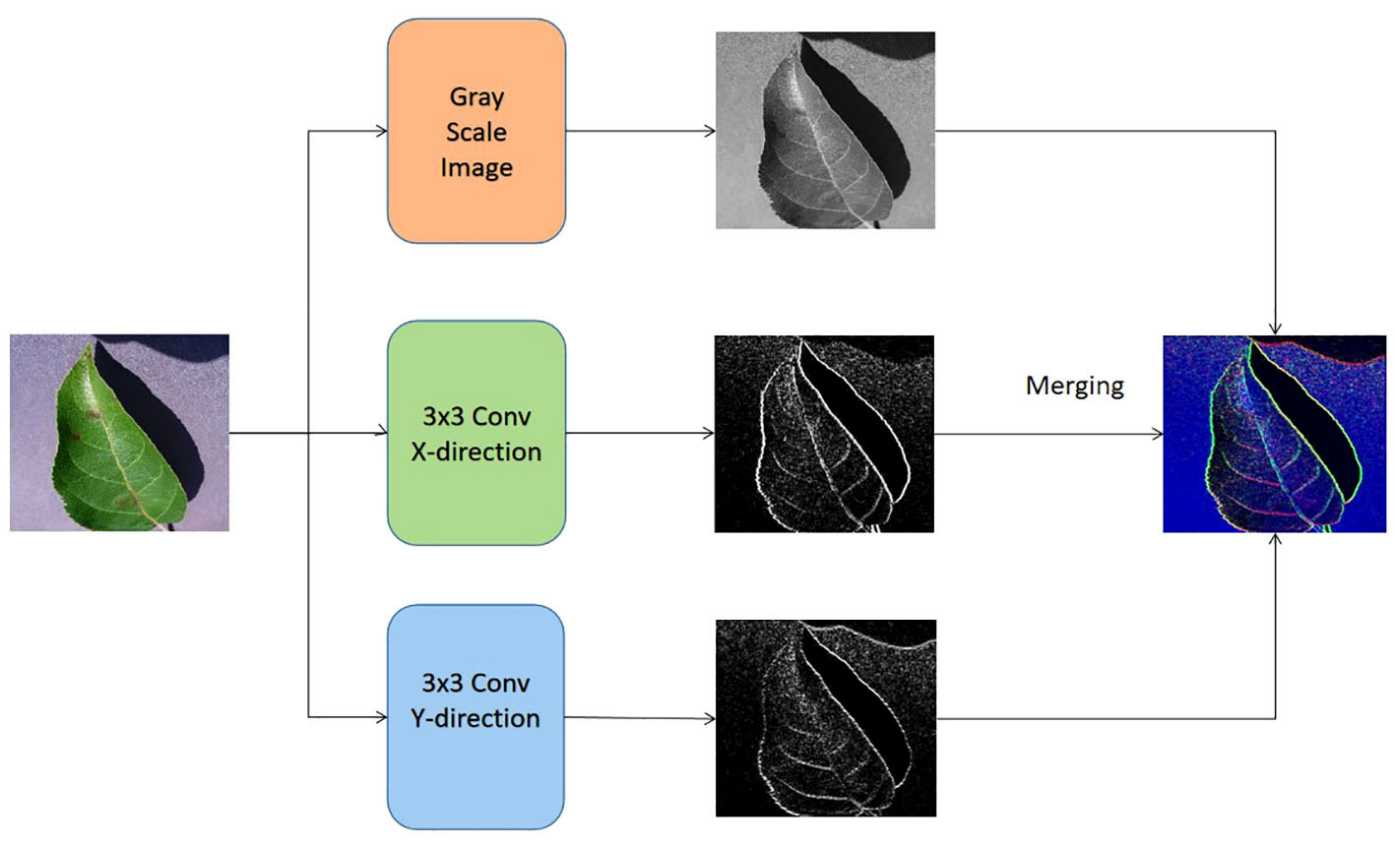
The formation of the input for the bottom channel of the proposed vision transformer model.


As seen in **Figure 3**, the input of the lower channel is achieved by the utilization of the Sobel operator Liu and Wang (2022) and the continuous image fusion operation. The edge Sobel operator is employed on the original image in order to provide input for the suggested methodology. Initially, the gray-scale equivalent is derived from each original image. Next, the original image undergoes convolution with the Sobel operators of size 3×3 in both the horizontal and vertical axes. The specific characteristics of the horizontal and vertical Sobel operators, denoted as *G_x_* and *G_y_* respectively, are outlined below in Equations 1 and 2.

(1) $$G_{x}=\left[\begin{array}{ccc} +1 & 0 & -1\\ +2 & 0 & -2\\ +1 & 0 & -1 \end{array}\right]\times I,$$

(2) $$G_{y}=\left[\begin{array}{ccc} +1 & +2 & +1\\ 0 & 0 & 0\\ -1 & -2 & -1 \end{array}\right]\times I,$$

where the original image is taken as I, and let *G_x_* be equal to the transpose of *G_y_*. It is worth noting that the elements in the operators *G_x_* and *G_y_* are differentiable. The starting values of the convolutional layer, also known as the Sobel operator layer, are determined by the elements in the *G_x_* and *G_y_* operators. These values may be optimized by a back-propagation approach during the training phase of the proposed transformer. In addition to combining the output of these two channels through concatenation, the classification process involves the utilization of a softmax classifier, an average pooling layer, and a fully-connected layer.

#### 2.2.1 Details of the backbone

As seen in **Figure 2**, the configuration of blocks in each channel and the size of tokens may be adjusted to accommodate diverse scales of machine vision applications. In accordance with the present investigation, the quantity of blocks in each channel is multiplied by a factor of 2, 2, 6, and 2, respectively. Following the input technique, the input image is initially partitioned into non-overlapping patches of size 4×4. Hence, the feature dimension of a single patch may be calculated as the product of its width, height, and number of color channels, resulting in a value of 48 (where 3 represents the number of RGB channels). In a manner akin to the vision transformer proposed by Dosovitskiy et al. (2020), the approach involves treating each patch as a token, where the feature representation of a token is obtained by concatenating the pixel values inside the associated patch. Different from the original vision transformer, the proposed transformer model leverages the swin transformer block and the shift-window self-attention mechanism.

In the initial stage, a linear embedding layer is employed to project the original feature into a dimension of arbitrary size (C=96 in the context of this work). Next, a series of swin transformer blocks are utilized on the tokens, incorporating two distinct forms of self-attention modules. Furthermore, it should be noted that the number of tokens in the swin transformer blocks stays consistent with the linear embedding unit, which is calculated as $\frac{H}{4}\times \frac{W}{4}$ .

The hierarchical representation is generated by the provided model through the utilization of patch merging modules, which effectively down-sample the feature resolutions by a factor of 2. The first merger module and feature modification are denoted as Stage 2, which are then repeated as Stage 3 and Stage 4. Furthermore, the dimensions of the output features progress from Stage 1 to Stage 4 as $\frac{H}{4}\times \frac{H}{4}\times C$ , $\frac{H}{8}\times \frac{H}{8}\times C,\;\frac{H}{16}\times \frac{H}{16}\times C$ , and $\frac{H}{32}\times \frac{H}{32}\times C$ , respectively. The hierarchical representation is primarily distinguished between the swin vision transformer Liu et al. (2021) and the original vision transformer Dosovitskiy et al. (2020) by the inclusion of Stage 2, Stage 3, and Stage 3 together. The given methodology does not include the utilization of any class taken. In this approach, the output vector of dimensions $N=\frac{H}{32}\times \frac{W}{32}$ is generated by using global average pooling followed by a fully-connected layer. The linear classifier then takes into account the first *C* components of this output vector.

#### 2.2.2 Swin transformer block

**Figure 4:**
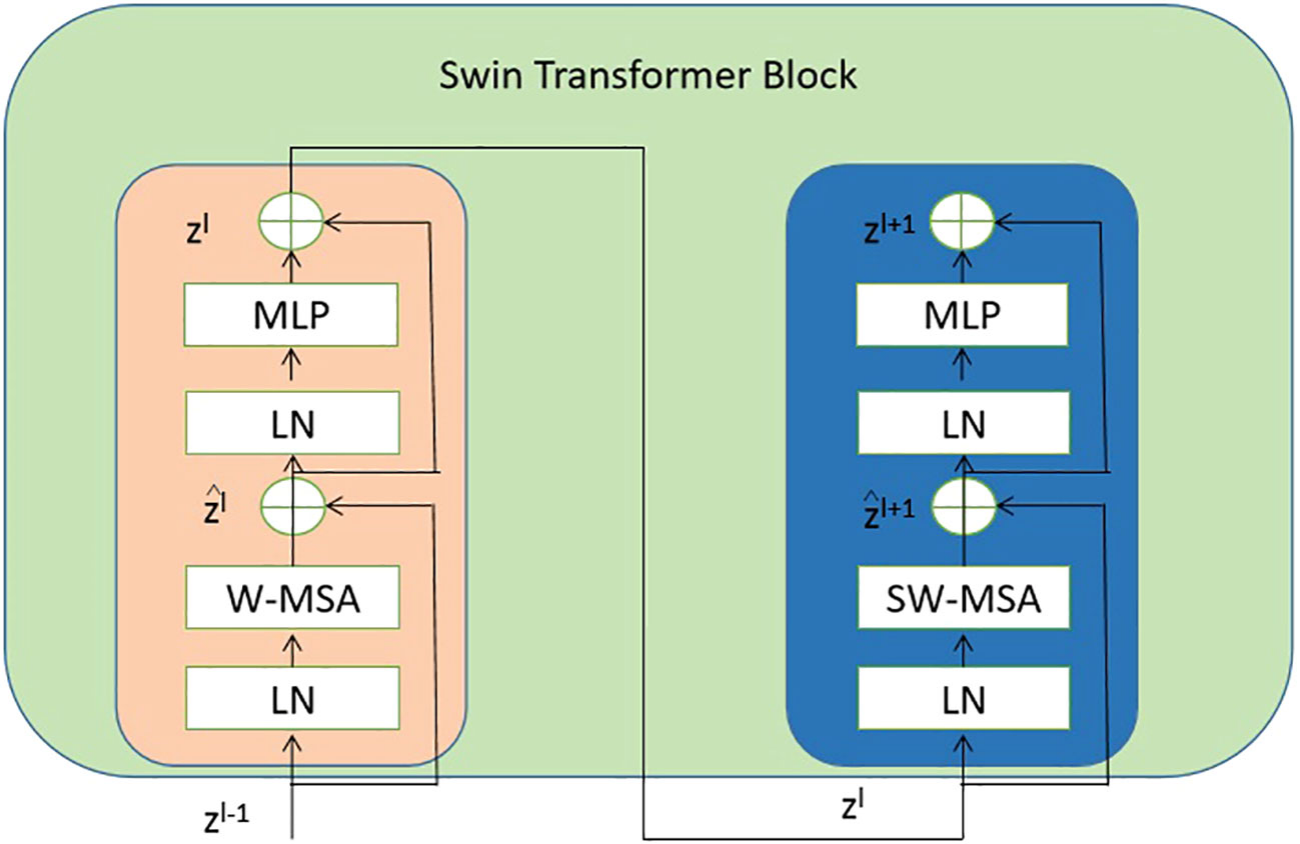
The detailed components inside the Swin Transformer model. The abbreviation LN is used to refer to layer normalization. The normal and shifted-windows multi-head self-attention modules are denoted as W-MSA and SW-MSA, respectively. The acronym MLP stands for multiple-layer perception. .

Each stage of the proposed model consists of the swin transformer blocks. And each swin transformer block consists of consecutive modules, as shown in **Figure 4**. In this architecture, there are two important modules W-MSA and SW-MSA, which represent the multi-head self-attention (MSA) with a standard window and the MSA with a shifted window, respectively.

The mathematical representation of the consecutive swing transformer modules can be articulated in Equations 3–6:


(3) $${\hat{z}}^{l}=W-MSA\left(LN\left(z^{l-1}\right)\right)+z^{l-1},$$

(4) $$z^{l}=MLP\left(LN\left({\hat{z}}^{l}\right)\right)+\hat{z},$$

(5) $${\hat{z}}^{l+1}=SW-MSA\left(LN\left(z^{l}\right)\right)+z^{l},$$

(6) $$z^{l+1}=MLP\left(LN\left({\hat{z}}^{l+1}\right)\right)+{\hat{z}}^{l+1},$$

where the notation W-MSA refers to window-based MSA, MLP stands for multiple layer perception Tolstikhin et al. (2021), SW-MSA represents shifted-window MSA, and LN signifies layer normalization Ba et al. (2016).

#### 2.2.3 Shifted window-based self-attention mechanism

In contrast to the initial vision transformer that heavily relies on global self-attention, which necessitates calculating the relationships between a token and all other tokens, the window-based MSA module employs a window of size *M* × *M* (with a default value of M=7) to restrict the extent of calculation. Hence, the computational complexity becomes more manageable with the incorporation of the window-based self-attention mechanism, as opposed to the quadratic complexity of the vision transformer Dosovitskiy et al. (2020), which is dependent on the image resolution *h* × *w* (as shown in Equations 7, 8).


(7) $$Ω\left(MSA\right)\;=\;4hwC2+2{\left(hw\right)}^{2}C,$$

(8) $$Ω\left(W-MSA\right)\;=\;4hwC^{2}+2M^{2}hwC,$$

where h and w denote the height and width of an image, C=96, and M=7 in the following settings.

**Figure 5:**
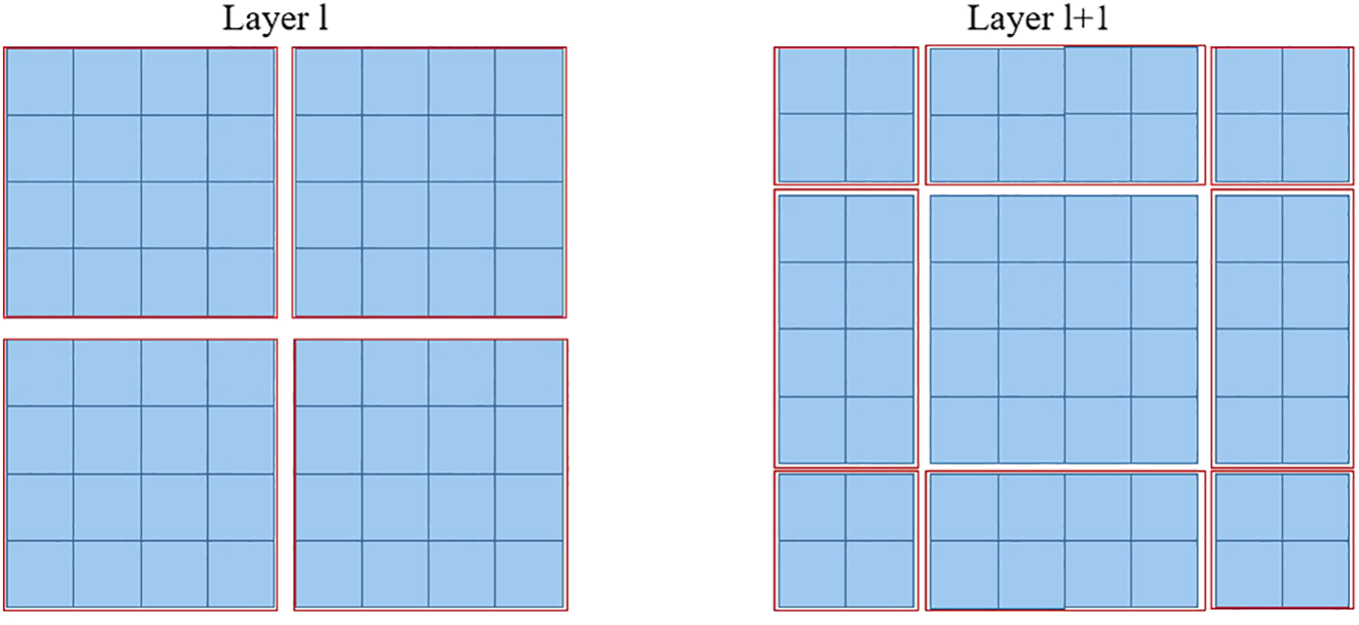
The diagram depicting the SW-MSA mechanism employed in the proposed methodology. The red boxes are used to indicate the local window, which serves the purpose of constraining the scope of self-attention calculation.

Furthermore, the SW-MSA strategy is intended to enhance the encoding of global relationships among the pixels in multiple windows. The use of the relationship across many windows may be maximized with the introduction of SW-MSA. As seen in **Figure 5**, the partitioning method of the regular window is employed in layer l, where self-attention is computed within each window. In the subsequent layer, denoted as l + 1, the partitioning of the window is adjusted both horizontally and vertically, resulting in the creation of a greater number of distinct windows. Thus, the self-attention calculation in Layer l+1 traverses the initial windows in Layer l.

It should be noted that the loss function employed in the proposed model is a cross-entropy loss. This loss is computed by comparing the ground truth category of the image with the classification output given by the suggested model, as seen in **Figure 2**.

## 3 Experiments

### 3.1 Implementation details

The tests were done employing four NVIDIA RTX 3080 GPUs, the PyTorch deep learning framework Paszke et al. (2019) version 2.0.1, and the Python programming language version 3.8.3. The backbone of the suggested model consists of the Swin-T vision transformer, which is employed for each channel. The dimensions of the input images are standardized to 224×224. Furthermore, the suggested swin vision transformer was initialized using the pre-trained weighting parameters of ImageNet Russakovsky et al. (2014). Typically, the hyper-parameters employed in the experiments encompass the subsequent elements, as shown in **Table 1** To note that the experiments by using the proposed approach were conducted in a 10-fold cross-validation scheme. Meanwhile, the hyper-parameters were determined by using a trial-and-error strategy.

**Table 1:** Hyper-parameters used in the experiments.

Item	Value
Batch_size	8
optimizer	Adam
learning rate	1e-4
depth	12
epochs	100

In order to assess the effectiveness of the suggested model and the comparison methodologies, the experiments contained several assessment measures, including accuracy, precision, recall, and F1 score (as shown in Equations 9–12).


(9) $$Accuracy=\frac{\left(TP+TN\right)}{\left(TP+TN+FP+FN\right)},$$

(10) $$Precision=\frac{TP}{TP+FP},$$

(11) $$Recall=\frac{TP}{TP+FN},$$

(12) $$F1=\frac{2\times Precision\times Recall}{Precision+Recall,}$$

where TP, TN, FP, and FN denote number of true positive, true negative, false positive, and false negative, respectively.

### 3.2 Ablation study

The proposed vision transformer model incorporates two distinct topologies for swin vision transformers. To evaluate the efficacy of the introduced swin vision transformer, a series of ablation experiments were conducted on a publicly available dataset. These experiments involved varying the settings of the introduced models, which were used to replace the original settings of the proposed approach. The original approach consisted of a vision transformer Dosovitskiy et al. (2020) and the Sobel operator with fixed 3×3 values.

**Figure 6:**
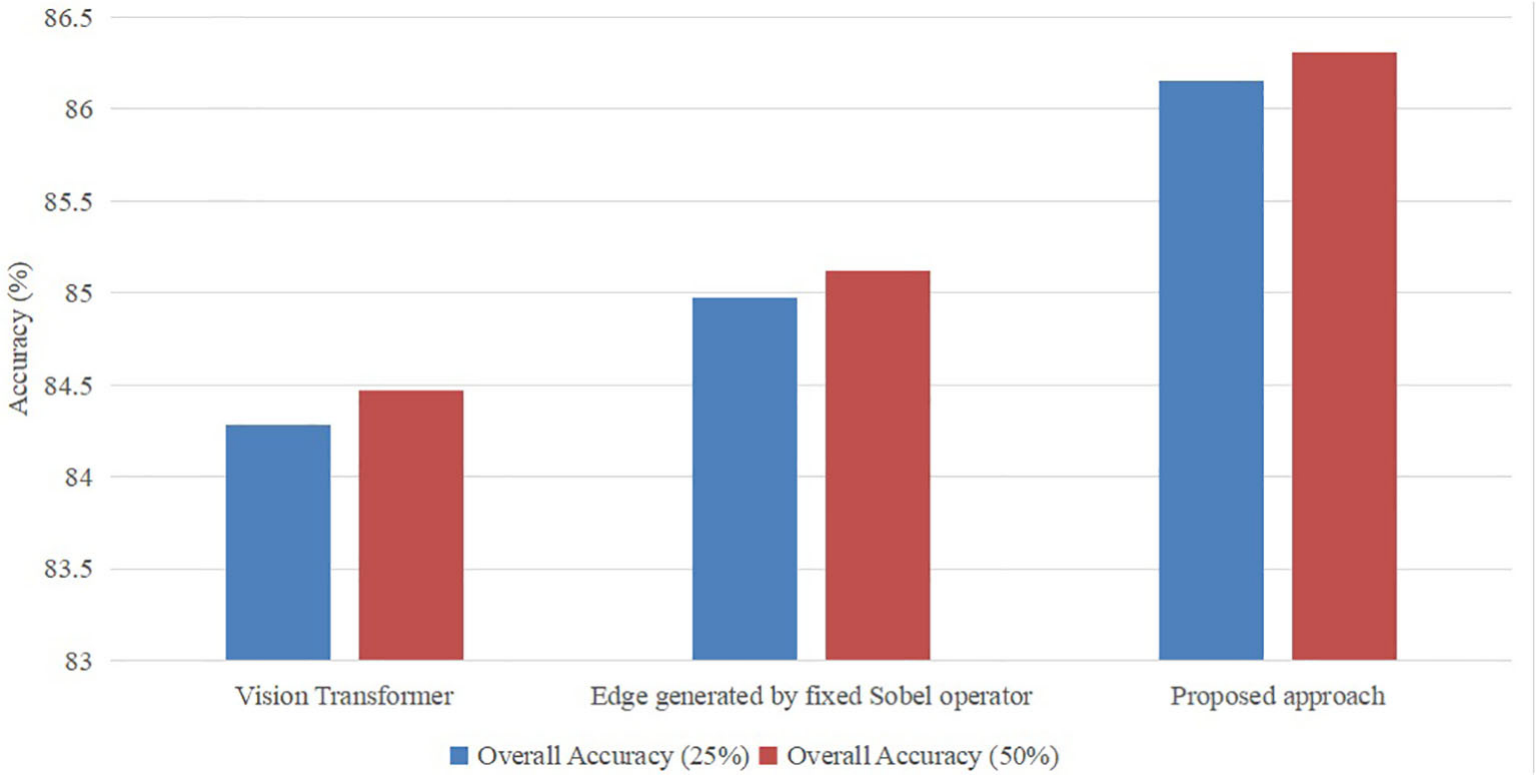
Ablation study with different settings with ratios (25% and 50%) of the training set in the publicly available dataset.

As seen in **Figure 6**, it is evident that the accuracy of the suggested methodology surpasses that of the model utilizing the original vision transformer or the fixed Sobel operator. The transformer model under consideration has demonstrated a performance improvement of 2.2% and 1.4% compared to the vision transformer version and the fixed Sobel operator version, respectively, when evaluated on a subset comprising 25% of the utilized dataset. Furthermore, the transformer model under consideration has demonstrated a performance improvement of 2.22% and 1.40% compared to the vision transformer version and the fixed Sobel operator version, respectively, when evaluated on 50% of the identical dataset. Hence, the selected model was deemed suitable as the foundational framework for the subsequent investigations.

### 3.3 Experimental results

To evaluate the performance of the proposed approach in a fair manner, the comparison experiments were conducted between the state-of-the-art methods, including, and ours on the same dataset as provided in **Table 2**.

**Table 2:** Distribution of the images in the dataset of this study.

Class	Label Name	No. of training images	No. of testing images
1	Apple healthy	1,185	169
2	Apple_Scab general	211	30
3	Apple_Scab serious	152	22
4	Apple Frogeye Spot	427	61
5	Cedar Apple Rust general	142	20
6	Cedar Apple Rust serious	40	6
7	Cherry healthy	598	85
8	Cherry_Powdery Mildew general	116	12
9	Cherry_Powdery Mildew serious	110	18
10	Grape healthy	294	42
11	Grape Black Rot Fungus general	381	54
12	Grape Black Rot Fungus serious	462	66
13	Grape Black Measles Fungus general	503	74
14	Grape Black Measles Fungus serious	419	59
15	Grape Leaf Blight Fungus general	61	9
16	Grape Leaf Blight Fungus serious	630	90
17	Citrus healthy	367	52
18	Citrus Greening June general	1,828	269
19	Citrus Greening June serious	1,799	262
20	Peach healthy	251	36
21	Peach_Bacterial Spot general	857	122
22	Peach_Bacterial Spot serious	770	110
–	Total	11,603	1,668

In order to objectively assess the performance of the proposed approach, a series of comparative experiments were conducted. These experiments involved benchmarking the proposed approach against several state-of-the-art methods, namely AlexNet Krizhevsky et al. (2012), GoogleNet Szegedy et al. (2014), VGG Abas et al. (2018), ResNet101 Zhang (2021), EfficientNetB3 Singh et al. (2022), Inception V3 Jenipher and Radhika (2022), MobileNet V2140 Elfatimi et al. (2022), and vision transformer Dosovitskiy et al. (2020). In the experiments, these state-of-the-art methods adopted their original settings in the literature. To note that the former seven state-of-the-art algorithms are CNN models. And the proposed approach was inspired by the work of the last model vision transformer. Meanwhile, the evaluation was carried out on the dataset specified in **Table 2**.

As seen in **Table 3**, the suggested strategy exhibits superior accuracy, precision, recall, and F1 score compared to existing state-of-the-art approaches. To provide specific results, our method demonstrates an increase in overall accuracy of 2.1% when compared to MobileNet V2140. Additionally, our proposed approach exhibits improvements in Precision, Recall, and F1 score by 2.6%, 2.7%, and 2.7% respectively, when compared to MobileNet V2140. Furthermore, even when compared to the original vision transformer, our approach showcases enhancements in accuracy, Precision, Recall, and F1 score by 1.1%, 1.5%, 0.59%, and 1.1% respectively. In summary, the suggested methodology demonstrates higher performance compared to both CNN-based and vision transformer-based algorithms. This provides evidence of the prospective capability of the proposed technique in feature extraction.

**Table 3:** Comparison results between the state-of-the-arts and the proposed method.

Method	Accuracy	Precision	Recall	F1 score
AlexNet	78.51	77.63	80.16	78.87
GoogleNet	81.23	80.59	82.05	81.31
VGG	82.35	82.19	82.94	82.56
ResNet101	83.18	82.56	83.29	82.92
EfficientNetB3	83.25	83.03	83.48	83.25
Inception V3	84.01	83.23	84.33	83.78
MobileNet V2140	84.69	83.52	84.92	84.21
Vision Transformer	85.47	84.38	86.71	85.53
Our method	**86.43**	**85.73**	**87.22**	**86.47**

Bold values denote the best performance.

**Figure 7:**
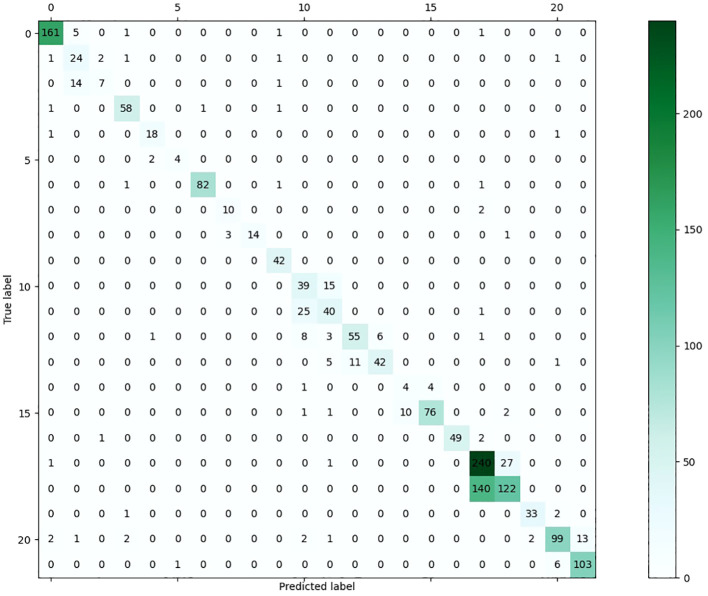
The confusion matrix of the proposed approach on the presented dataset.

In order to assess the effectiveness of the suggested methodology on various image categories within the leveraging dataset, we have included the accuracy-based confusion matrix (as seen in **Figure 7**) for the proposed technique. This matrix pertains to the 22 categories of leaf disease images inside the public dataset. The majority of the categories have demonstrated encouraging outcomes. The leaf disease images that exhibit inadequate classification pertain to the plant species “Apple” and “Citrus.” The category labeled as “Citrus healthy” can sometimes be mistaken with the category known as “Citrus Greening June general.” The attribution of the resemblance between various forms of leaf diseases is warranted. Another challenging classification assignment involves distinguishing between “Apple_Scab general” and “Apple_Scab serious.” This phenomenon may be ascribed to the existence of two distinct variants of an image falling under the overarching classification of “Apple_Scab.”

**Figure 8:**
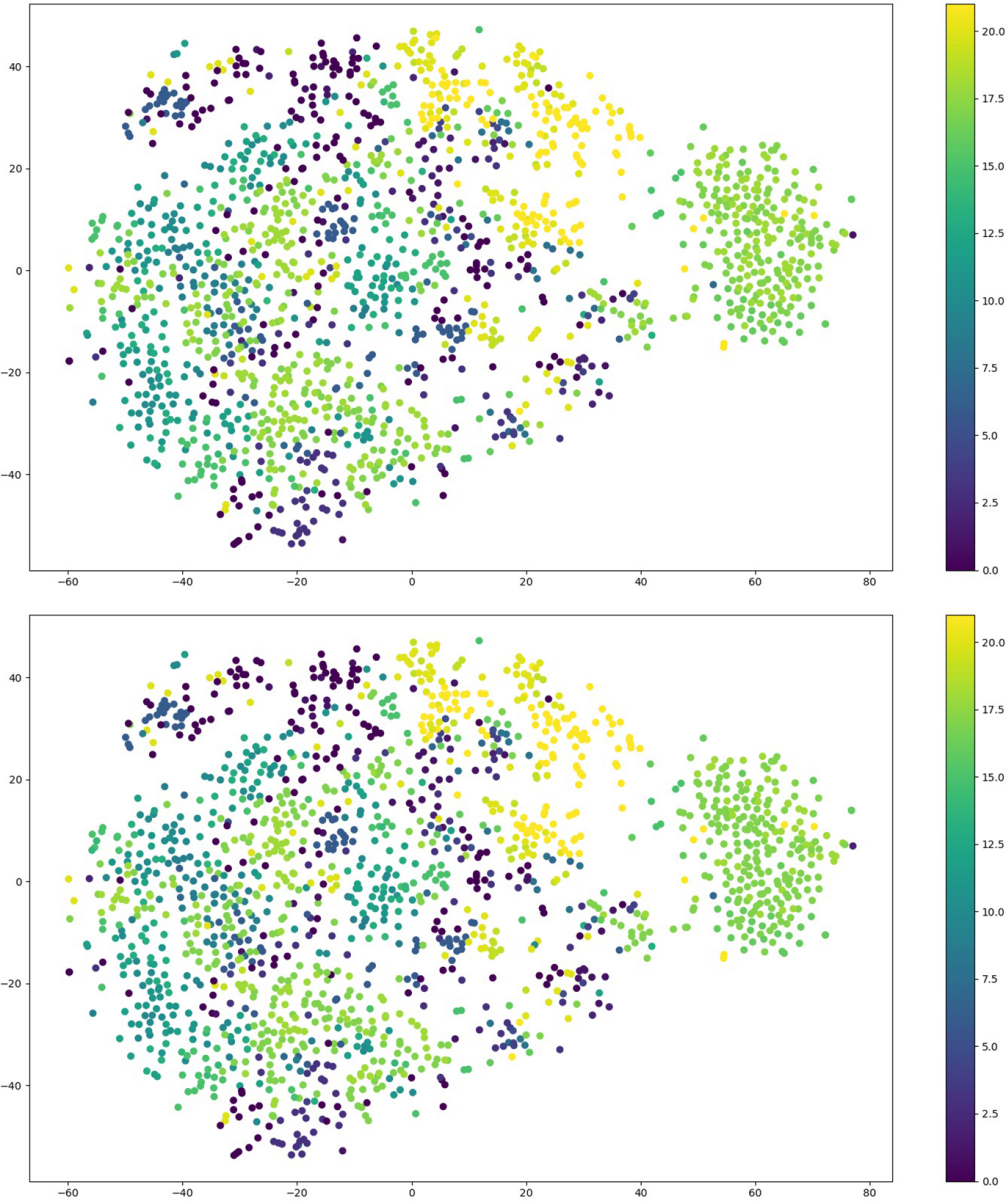
The outcome of performing t-SNE on outcome generated from the proposed approach (Top) and on the ground truth samples (Bottom).

In addition, the T-distributed stochastic neighbor embedding (t-SNE) was implemented using the suggested methodology, as seen in **Figure 8**, van der Maaten and Hinton (2008). It should be noted that t-SNE is a computational approach employed for the purpose of visualizing the multidimensional feature space of the 22 categories of sick leaves in a two-dimensional (2D) format. **Figure 8** presents a summary of the t-SNE clustering outcomes for both the output produced by the suggested technique and the ground truth. **Figure 8** exhibits a notable clustering pattern as classes 16 and 17 are closely packed together on the right side. It should be noted that the distinct attributes of these leaf images can only be ascribed to a limited number of locations that are outside the clusters.

### 3.4 Discussion

The utilization of CNN models in deep learning has become prevalent. These models possess the capability to extract feature maps from images. Furthermore, the effectiveness of feature extraction may be enhanced by employing a network structure with increased depth. Nevertheless, the efficacy of CNNs may be limited due to the inherent constraint of the convolutional module, which primarily emphasizes the analysis of small receptive fields inside the images. This phenomenon rapidly results in the disregard of the interconnections among distant pixels within an image. In addition, the process of enhancing the performance of deeper convolutional neural network models necessitates a greater allocation of processing resources.

**Figure 9:**
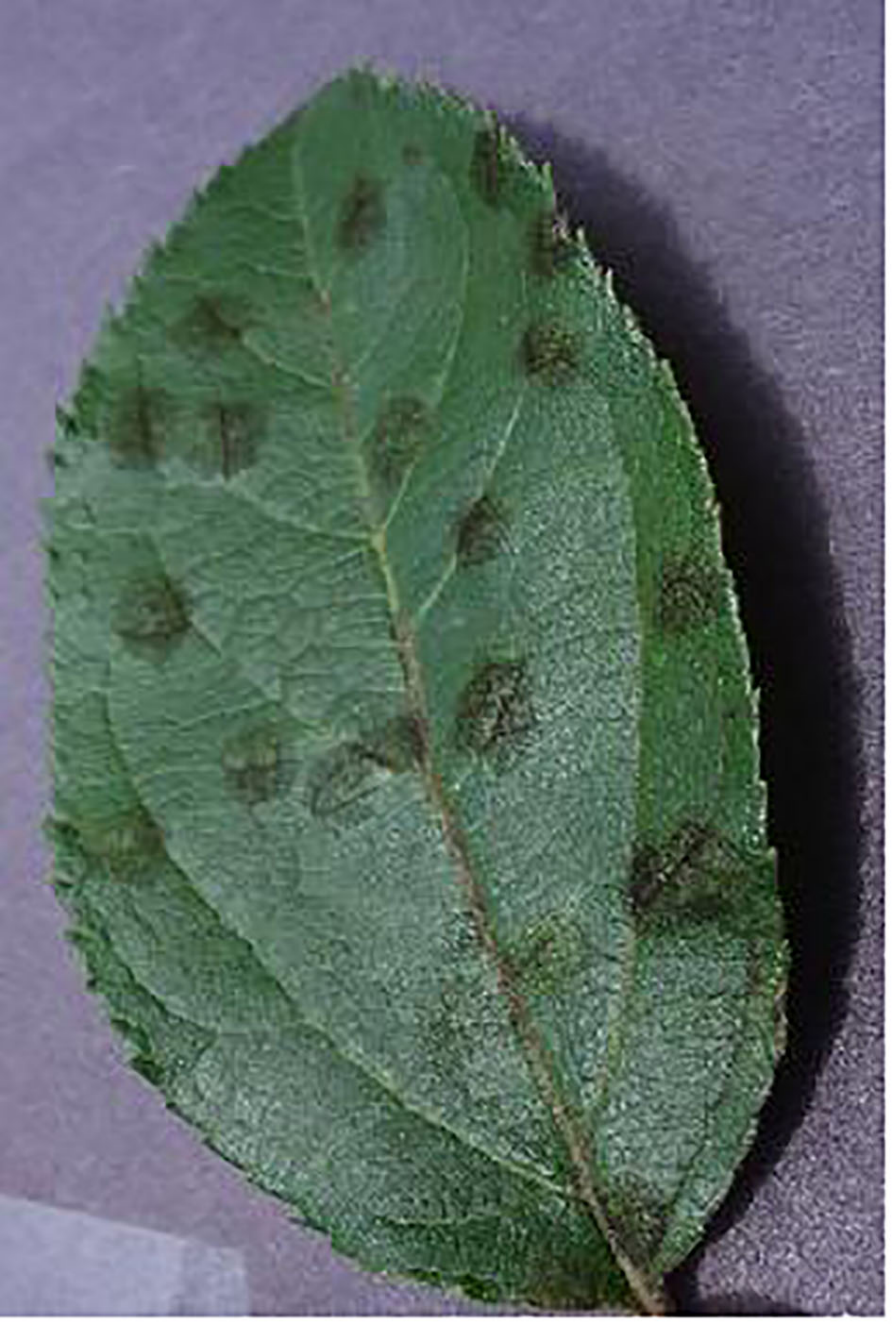
A sample image contains the diseased areas across the entire image.

In the context of leaf disease images, it is observed that the affected regions are frequently dispersed over the whole image, rather than being confined to a specific localized location. This characteristic is exemplified in **Figure 9**. Given the limitations of the local receptive field mechanism in addressing the specific leaf disease image, the mere addition of extra layers to the CNN models does not always ensure improved performance in image classification. This study presents the introduction of a vision transformer-based model for image classification, which leverages the relationships among distant pixels inside the images. The suggested dual channel model employs the technique of MSA to continually extract the correlation between image patches. This approach effectively preserves the information that is advantageous for classification purposes. In contrast to the original vision transformer model, the swin vision transformer model is capable of extracting valuable information from images while concurrently mitigating its computing resource requirements. Nevertheless, this research endeavor is subject to many constraints: The dataset utilized in the experiments suffers from unbalanced image samples, hence limiting the effectiveness of the presented method. Meanwhile, number of the image samples contained in the leveraged dataset is still limited, which constrains the accuracy of the proposed approach at relatively low level. In addition, there exists duplication between the edge information included in the lower channel of the proposed model and the upper channel.

## 4 Conclusion

The present study introduces a novel network architecture for leaf disease image classification, utilizing a two-channel swin transformer-based approach. The system consists of a dedicated channel for the original image and an additional channel specifically intended to capture the edges in the merged image. In addition, the Sobel operator has been utilized to extract the edge information from the images of leaf diseases. The utilization of the two-channel swin vision transformer model has resulted in the attainment of improved performance compared to the current state-of-the-art methods. The efficacy of the suggested model is demonstrated by experimental findings conducted on the publically accessible dataset. The experimental results of the proposed approach have proved the superior performance of the proposed approach in leaf disease classification. It can be concluded that the proposed approach could be a valuable algorithm for leaf classification and Precision Agriculture.

Recently, there has been encouraging performance demonstrated by vision transformer-based models in challenges related to multi-modal machine vision. Henceforth, we shall further explore the intricacies of multi-model-based deep learning models in the context of leaf disease categorization and prediction. In addition, more samples need to be collected to eliminate the class imbalance issue in the dataset used in this study.

## Data availability statement

Publicly available datasets were analyzed in this study. The datasets for this study can be found in the AI challenger 2018 at https://aistudio.baidu.com/datasetdetail/76075.

## Author contributions

DH: Conceptualization, Data curation, Formal Analysis, Investigation, Visualization, Writing – original draft. CG: Funding acquisition, Methodology, Project administration, Supervision, Validation, Writing – original draft.
